# Why workshops work: Examining the efficacy of training trainers to train goats

**DOI:** 10.1017/awf.2023.94

**Published:** 2023-11-21

**Authors:** Jennifer Meier, Viviane Theby, Lorenz Gygax, Edna Hillman, Carola Fischer-Tenhagen

**Affiliations:** 1 German Federal Institute for Risk Assessment (BfR), Centre for the Protection of Laboratory Animals (Bf3R), Berlin, Germany; 2 Tierakademie Scheuerhof; Wittlich, Germany; 3 Humboldt-Universität zu Berlin, Faculty of Life Sciences, Albrecht Daniel Thaer-Institute of Agricultural and Horticultural Sciences, Animal Husbandry & Ethology, Berlin, Germany

**Keywords:** animal trainer education, animal welfare, clicker training, husbandry training, refinement, ruminants

## Abstract

Experimental procedures involving farm animals are often associated with stress due to restraining. Stress can be reduced through use of positive reinforcement training, which then serves as refinement according to the 3Rs principles. Trainer skills, however, may influence the feasibility and success of animal training. The potential influence of trainer skills as well as the education of animal trainers are rarely described in literature but are necessary information for the implementation of positive reinforcement training as a refinement measure. To investigate the effect of educational programmes on animal trainers, we compared the training success of two groups of participants in training goats to elicit a behaviour that would allow simulated venipuncture. One group was educated in a two-day workshop while the other was provided with specific literature for self-instructed learning. Training success was evaluated using an assessment protocol developed for this study. A greater training success in the WORKSHOP GROUP, reflected by objective and subjective measures, was clearly supported statistically. In addition, 73 versus only 13% of the participants of the WORKSHOP GROUP and the self-instructed BOOK GROUP, respectively, stated that they could completely implement the knowledge gained in the course of this study. Our results indicate that more intensively educated trainers can train animals more successfully. In conclusion, if animal training is implemented as refinement, animal caretakers should receive instruction for positive reinforcement training.

## Introduction

Fewer farm animals are used in (biomedical) research than rodents, but a fair number of these animals are regularly subjected to experimental procedures. In 2020, 118,002 mammalian farm animals, such as pigs, goats, sheep, cattle, and equids, were used for experiments in the EU, including Norway (European Commission 2023). Experiments with farm animals include basic research, translational and applied research, as well as regulatory use and routine production. With further progress in xenotransplantation (Hawthorne 2022), which describes the transplantation of organs, tissue or cells from one species (e.g. pig) to another (e.g. human), and numerous examples for farm animals as biomedical models (Hamernik 2019), their relevance may increase in the future.

Special caution is required when handling farm animals due to their size and strength. Forceful restraining techniques are often implemented for job safety and practicability. These techniques include manual restraint, livestock crushes, or headlocks. Certain techniques (for example, the use of a nose plier in cows) restrain the animal and distract it simultaneously by inflicting modest pain (Rosenberger *et al.* 1977). Various studies have shown that routine procedures, for instance separation, blood sampling or restraining techniques can cause stress in different species of animals (Balcombe *et al.* 2004; Yardimci *et al.* 2013; do Vale *et al.* 2020). Stress, in turn, affects the welfare of the animals involved and may also affect the reliability of the results in those studies (Poole 1997; Bailey 2018).

Reducing stress in laboratory animals to an absolute minimum reflects refinement according to the 3Rs principles (Russell & Burch 1959). Accordingly, refinement is thought to increase animal welfare. In the desire to strike a balance between reducing animals’ stress while ensuring procedures are able to be performed, both in terms of safety and practicality, a solution may exist in the form of husbandry training. Here, we define husbandry training as animal training based on shaping through positive reinforcement with the intention of encouraging the animal to co-operate and voluntarily endure husbandry and/or veterinary procedures. The term shaping was introduced by Skinner (1951) and describes the selective reinforcement of gradually improving approximations to a specific desired response or behaviour. Some examples in shaping of farm animals are trailer-loading for horses (Ferguson & Rosales-Ruiz 2001; Slater & Dymond 2011) or sheep shaped to behave like petting sheep (Fernandez 2020). Shaping involves so-called reinforcers. A primary positive reinforcer is a reward (e.g. food) following a behaviour, which increases the likelihood of the animal repeating said (desired) behaviour. This stimulus is regularly presented after the occurrence of the behaviour. A clicker works as a so-called secondary reinforcer. A secondary reinforcer is a signal, such as a sound or spoken word, that serves as a time-bridge between a behaviour and the pleasant stimulus (primary reinforcer).

Evidence exists of positive effects of husbandry training (Schapiro *et al.* 2001; Laule *et al.* 2003; Bloomsmith *et al.* 2015; Leidinger *et al.* 2017; Lomb *et al.* 2021). For instance, Schapiro *et al.* (2001) describe that positive reinforcement training increases affiliative behaviour among rhesus macaques (*Macaca mulatta*), which may indicate a general increase in welfare through training. Furthermore, Lomb *et al.* (2021) demonstrate that positive reinforcement training reduced avoidance behaviour in cattle during and after subcutaneous injections. Husbandry training conducted with positive reinforcement has its origin in zoos (Fernandez & Martin 2021) and has become an essential component of work with zoo animals (Colahan & Breder 2003; Savastano *et al.* 2003; Bloomsmith *et al.* 2015; Melfi *et al.* 2020). However, it has rarely been described for farm animals. Some of the few examples with farm animals are sling (Jonholt *et al.* 2021) or target training (Yang *et al.* 2021) in pigs, or training for subcutaneous injections in cattle (Lomb *et al.* 2021).

Animals can be trained with varying efficiency in respect to invested time and precision of the trained behaviour (Schapiro *et al.* 2003; Paredes-Ramos *et al.* 2020). Schapiro *et al.* (2003), for instance, review studies on the effectiveness of positive reinforcement training with non-human primates focusing on the time required for training and different training procedures. However, the influence of the animal trainer and their education on training efficiency is not discussed (in this text, we use they/them/their as gender-neutral terms). Since animal training is an interaction between animals and a human, the animal trainer and their skills may heavily influence the training outcome. Training skills include, e.g. the general knowledge of animal training and learning, the precision in timing of the secondary reinforcer, and the structured implementation of a training schedule (Sevenich‐MacPhee 2019). A training schedule outlines each step of the training (e.g. as described in Leidinger *et al.* 2017). However, we are not aware of any previous studies on specific skills necessary for an animal trainer and educational programmes for animal trainers are seldom described (Lukas *et al.* 1998; Sevenich‐MacPhee 2019). This makes it difficult to compare animal training between studies as shown by Johnen *et al.* (2013). Yet, we assume that skills and education of trainers are essential for the implementation of husbandry training as refinement and, in turn, an increase of animal welfare in farm animals. Furthermore, we see the potential for improved animal welfare through husbandry training as enrichment (Fernandez 2022) and as a tool to improve a positive human-animal relationship (Rault *et al.* 2020).

In Germany, animal training is not part of the curriculum of animal caretakers, technicians or veterinarians. Training skills have to be acquired ‘on the job’. Due to the limited time and resources of personnel, education needs to be efficient in respect to duration and effectivity. Several studies show that practical approaches and hands-on training lead to improved learning compared with purely theoretical instructions in husbandry training (Miotto *et al.* 2010; Bansal & Aggarwal 2014).

To our knowledge, this is the first study aiming to investigate the potential influence of two different ‘train the trainer’ programmes on the success of training an animal. For this, we compared the training success of two differently educated groups of persons in training goats to show a behaviour allowing simulated venipuncture. One group was educated in a two-day workshop (WORKSHOP GROUP), the other studied on their own with the help of a book on positive reinforcement training (BOOK GROUP). We hypothesised that those participants instructed in a workshop would have greater training success compared to those self-instructed by a book.

## Materials and methods

### Ethical statement

The study was conducted in accordance with the guidelines of the German Animal Welfare Act and approved by the Berlin State Authority (‘Landesamt für Gesundheit und Soziales’, permit number: StN 0018-20). The study was not considered animal experimentation in the true sense of the law since no pain, suffering, or injuries were expected. In respect to the human participants, it was approved by the Ethics Committee of the Thaer-Institute for Agricultural and Horticultural Sciences at the Humboldt-Universität zu Berlin (permit number 2021-01).

### Human participants

We recruited the participants via various announcements including a short description of the project. Information was distributed through social media groups of veterinary students of the Freie Universität Berlin (FU Berlin; on Facebook), and dog owners (WhatsApp), as well as in agricultural science lectures at HU Berlin (by EH), or in direct contact with private acquaintances (of JM). The only inclusion criteria were availability for the date of the workshop, and the subsequent 14 days of training. No previous experience in animal training was requested, but such experience was not used as an exclusion criterion either. We registered 36 interested persons on a waiting list and finally included 31 participants (on a ‘first come, first serve’ basis) corresponding to the available number of goats. The participants were randomly allocated into two groups: the WORKSHOP GROUP (n = 16) and the self-instructed BOOK GROUP (n = 15) and matched with one specific goat each (using the Excel function RANDBETWEEN; Microsoft Excel® 2018). In total, 30 participants (15 in each group) joined the training period. One person from the WORKSHOP GROUP dropped out after the workshop without stating a reason. All participants either owned at least one pet at the time of the study or had done so previously. The majority of participants worked in an animal related job (n = 18/30) and had prior education and/or experience with animal training (Table 1). Approximately one-third stated prior experience in the use of a secondary reinforcer. There were no prominent differences in previous experience between either group.

**Table 1.** Experience of the participants (n = 30) in animal training assessed by the initial questionnaire for the two educational programmes (WORKSHOP/BOOK GROUP) and in total

		Workshop Group	Book Group	Total	
Prior education	both	7	4	11	with prior
“Have you previously gathered any information about animal training”	practical	1	1	2	education:
	theoretical	2	7	9	22
	none	5	3	8	
Experience years	> 10 yrs	3	3	6	with prior
“I have experience in animal training: amount of time…”	> 5 yrs	2	1	3	experience:
	> 1 yrs	2	3	5	20
	< 1 yr	2	4	6	
	none	6	4	10	
Experience with animals	>10	2	1	3	with prior
“How many animals have you already trained?”	6-10	0	1	1	animals
	2 to 5	4	6	10	trained:
	1	4	3	7	21
	none	5	4	9	
Prior experience with a	yes	5	6	11	
secondary reinforcer	no	10	9	19	
“Have you previously used a secondary reinforcer during animal training?”					

### Study animals and housing

**Figure 1.**
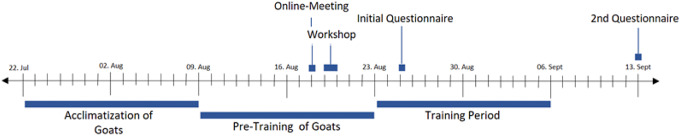
Time-line of the project.

**Figure 2.**
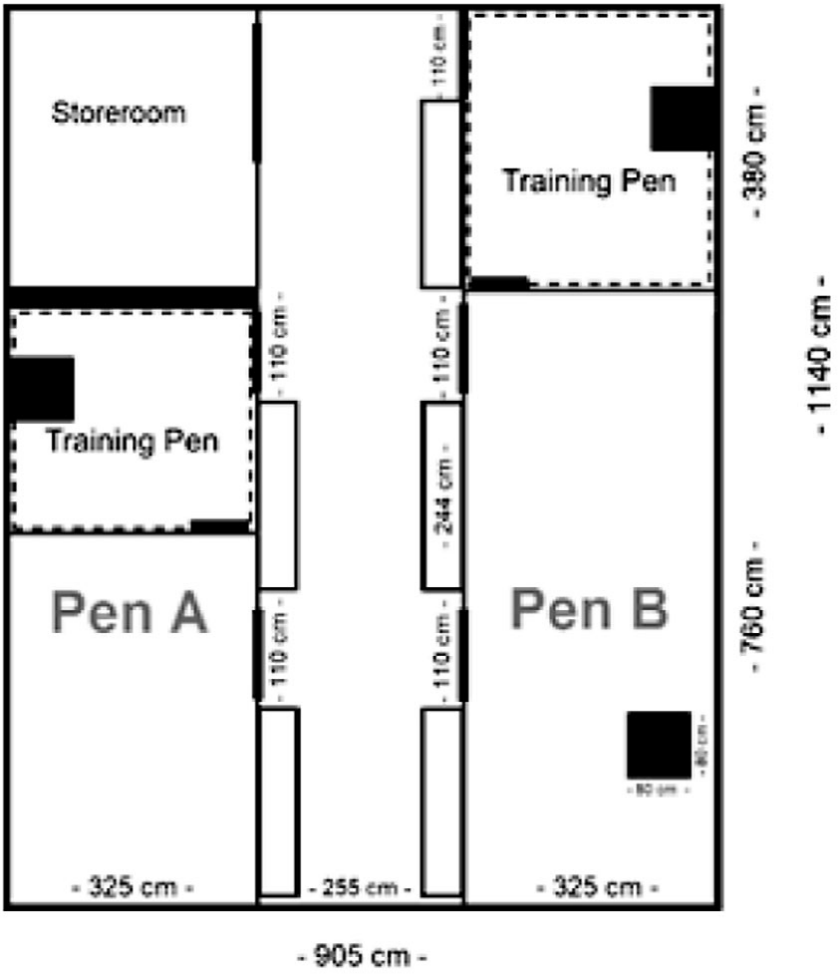
Layout of the barn. Nine female goats were housed in pen A and ten female goats in pen B. The male goats (n = 13) were housed in an identical barn in the same type of pen as pen B. In the barn for the males, the training pen of Pen A was also used for their training. Black squares represent platforms; rectangles in the alley represent feeding troughs.

Thirty-two mixed breed goats were included (White German, Thuringian, and Peacock goats; 19 females, 13 castrated males) that had been bred at the Teaching and Research Station for Farm Animal Sciences (HU Berlin, Germany). They were aged five to six months and weaned upon arrival at the experimental farm at the German Federal Institute for Risk Assessment (BfR). Thirty-one goats were randomly allocated to the participants; one randomly selected goat was trained by VT as an expert reference standard prior to the workshop. Prior to arrival at the BfR, the goats were managed under standard farming conditions with no specific handling other than feeding, vaccination (once), weighing, or cleaning the pen at HU Berlin. The goats arrived at the BfR four weeks before the training period started (Figure 1) and were housed indoors on straw in groups of 13 males (on 37 m²) as well as ten (on 37 m²), and nine females (24.7 m²; Figure 2). In each pen, a transparent fence with a small gate separated a training area from the rest of the pen. The training areas each included one platform measuring 78 × 78 × 78 cm. Apart from during training sessions, goats had free access to the training areas as well as *ad libitum* access to hay (first cut, foliate), provided in hay bags and in troughs, stocked-up twice a day, mineral salt blocks, and fresh water. Treats, such as concentrated feed, uncooked pasta, apples, twigs of beech or birch, were provided during the pre-training period (only by staff) and during the training period. Participants were allowed to use their own favourite treat as long as it was compatible with goats. Concentrate feed, pasta and twigs were provided at the training site. A maximum of 500 g of treats per animal per day were given to prevent acidosis. After two weeks of acclimatisation, BfR-staff habituated the goats to taking treats from their hands. This took place over a period of two weeks, once daily, for approximately 30 min per pen (pre-training period; Figure 1).

### Study design

**Figure 3.**
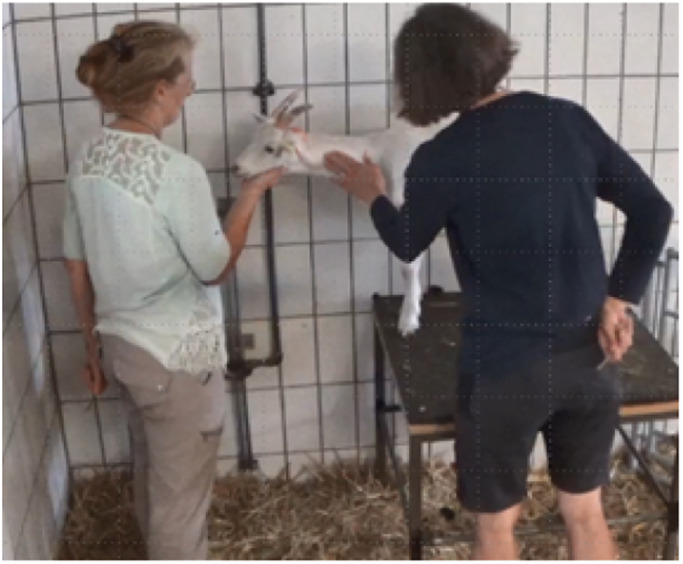
Target behaviour: Goat is standing still on platform, chin laid on trainer’s hand, and is tolerating being touched on the neck by a second person. Demonstrated by Viviane Theby (VT) as the expert reference standard.

In the week prior to the onset of the training period, all participants received information about the organisational aspects of the training and the training goal (target behaviour) in an online meeting (Figures 1 and 3). After this meeting, the same information was delivered in the form of a written document. All participants were aware of the outline and objective of the study.

The target behaviour was described as follows: (1) Goat jumps onto a platform and stays standing still; (2) Goat lays its chin on the participant’s hand and holds this position; (3) Goat tolerates being touched in the area of the jugular vein with light pressure by a second familiar person (staff member).

### Expert reference standard

The target behaviour was trained with an exemplary goat by VT. We defined VT’s training outcome as the expert reference standard due to her experience. VT is a veterinarian with an additional qualification in behaviour therapy, a diploma in animal behaviour counselling, and over 20 years of experience in animal training and the education of animal trainers.

### Workshop group

Participants in the WORKSHOP GROUP attended a two-day workshop at the BfR, Berlin. One of the authors (VT) taught the workshop. The workshop was composed of theoretical input, lasting approximately 9 h, and practical parts, including role-plays, lasting approximately 3 h. The theoretical input covered the subjects (positive) reinforcement, learning theory, as well as classical and operant conditioning. Additionally, the use and timing of positive reinforcement, body language, and the development of a training schedule were practiced among the participants. Finally, participants practiced positive reinforcement training with a secondary reinforcer on sheep. Here, they specifically focused on the training of the chin position on the trainer’s hand. The development of a training schedule, as well as the training on sheep were case-based education tailored to prepare for the training of the target behaviour. Case-based education imparts general knowledge through specific scenarios (cases).

### Self-instructed group (book group)

A week prior to the start of training, participants were given the book, *Verstärker verstehen* (Theby 2020) which included all the basic information given in the workshop. Participants were also encouraged to seek further information using any other resources, e.g. the internet.

### Training

In seeking to train their goat, participants were able to book as many time-slots as desired during the two-week training period and an online booking system consisting of 45-min time-slots was set up for participants. Slots could be booked between 0900 and 1800h and each 45-min slot included time for preparation (changing clothes, moving the goat to the training pen) and 30 min of training with the goat. Participants could stop each training session at any time. Each slot allowed only one participant per training pen, with a maximum of two participants per barn during the same slot. In 76% of the training slots used, only one participant trained their goat per barn, whereas in the remaining 24%, two participants trained their goats simultaneously. The participants were given no instructions on how to structure their training nor how to train the target behaviour. Two to three training sessions per week were recommended, but not obligatory. Participants used between two and ten time-slots for training and each session was video-recorded (Sony HDR-CX240E Handycam, Sony Tokyo, Japan). The camera was fixed on a flexible tripod on a rail of the pen partition and a staff member was always on hand to set up the camera, start the recording, and set an alarm to notify each 30 min. They also responded to any organisational questions and would touch the goat if participants so requested (target behaviour step 3). The member of staff did not help with training nor offer any advice. Additionally, participants were asked not to talk about the training with each other.

### Survey on experience and training success

The start of the training period saw participants asked to complete an initial questionnaire reporting information on their age, gender, occupation, ownership of pets, and previous animal training experience (Figure 1, Table 2). A week after the training period, participants received a second questionnaire which included questions regarding a self-assessment, and an evaluation of the training and the corresponding educational programme (Figure 1, Table 2).

**Table 2.** Names, definitions, and coding of the predictors and outcome variables collected by questionnaires and video analysis. Initial questionnaire as the basis for the predictor variables and 2nd questionnaire (self-assessment) as well as assessment protocol for video analysis as the basis for the outcome variables

Variable	Definition	Coding
Predictors (initial questionnaire)		
Educational programme	Workshop or Self-Instructed	1 / 0 = WORKSHOP / BOOK GROUP
Sex of goat		1 / 0 = female / male castrated
Age	Age of participant	5 / 4 / 3 / 2 / 1 = >40 / 34-40 / 26-33 / 18-25 / <18
Job with animal	Do animals play a role in the participant´s profession?	1 / 0 = yes / no
Prior education	Did the participant have prior education in animal training?	3 / 2 / 1 / 0 = both / practical / theoretical / none
Experience years	How many years of experience in animal training did participant have (self-assessment)?	4 / 3 / 2 / 1 / 0 = >10 yrs / >5 yrs / >1 yr / <1 yr / none
Experience with animals	How many animals did the participant train before (self-assessment)?	4 / 3 / 2 / 1 / 0 = >10 / 6-10 / 2-5 / 1 / none
Video analysis (assessment protocol, see Supplementary material **)**		
Session [#]	The total number of sessions conducted	
Duration [min]	Total duration of training sessions	
Jump	Grade (1-6) for “goat on platform” (element I in the Supplementary material	1 = equivalent to expert reference standard 2 to 5 = deviations from expert reference standard 6 = not showing any of the target behaviour
Chin	Grade (1-6) for “chin on hand” (element II)	see jump
Touch	Grade (1-6) for “touch of the neck” (element III)	see jump
Standing still	Grade (1-6) for standing still (element IV)	see jump
Trait trainer	Grade (1-6) for behaviour of participant (element V)	see jump
Respectfulness	Grade (1-6) for respectfulness of the goat (respectful goat: keeps a certain distance from the human and does not make body contact; element VI)	see jump
Goal achieved	Overall grade (1-6) for achieving the three tasks (jump, chin, touch; element VII)	see jump
Self-assessment (2^nd^ questionnaire)		
Time slots	“The offered training slots were sufficient for me”	1 / 0 = yes / no
Former experience	“I mostly trained based on my former (training) experiences”	1 / 0 = yes / no
Personal instruction	“I would have trained more effectively with individual instruction”	1 / 0 = yes / don’t know or no
Implement content	“I was able to implement the content of the book / workshop during training”	2 / 1 / 0 = completely / partly / not at all
Content use helpful	“The implementation of the book’s / the workshop’s content improved my training”	3 / 2 / 1 / 0 = yes / rather yes / rather no / no or did not implement the content
Goal self-assessment	“I accomplished the training’s task”	1 / 0 = yes / no
Chin self-assessment	“The goat laid its chin calmly on my hand”	1 / 0 = yes / no
Questions of 2^nd^ Questionnaire for descriptive evaluation		
“What was the training goal?” Complete description of goal included: “calm” OR “stand still” AND “jump on platform” AND “chin in hand” AND “touch of neck”		1 / 0 = complete/ incomplete description
“Training goals were achievable”		1 / 0 = yes / no
“Training goals were clear to me”		1 / 0 = yes / no
“Available time slots were sufficient”		1 / 0 = yes / no
“I created a training schedule”		1 / 0 = yes / no

### Data recording

To evaluate the relative success of the training, a specific assessment protocol was created (see Supplementary material). A video sequence showing a goat (trained by VT and touched by CFT) performing the target behaviour was defined as the expert reference standard (Figure 3). We then defined criteria to assess how well this expert reference standard was met by the participants. This was based on a score including six grades (as in German schools), with ‘1’ equivalent to the expert reference standard and ‘6’, if the goat did not show the desired behaviour at all (Supplementary material). The protocol was provided to two observers (students of agricultural science), who had no contact with the participants. JM trained these observers to evaluate video sequences based on short sample videos (taken from the training period of the goats). These sequences were not included in the final analysis. Further adjustments were made to the protocol, with more distinct descriptions included where definitions had not been clear to the observers.

Prior to the analysis of the final videos, we tested the inter-observer agreement of our assessment protocol. To do so, we used the Kendall’s coefficient of concordance (R package ‘irr’; Gamer *et al.* 2019). The data used for this assessment consisted of the evaluation of six test video sequences with 12 criteria each graded by the three observers (6 × 12 = 72 grades for JM and the two HU students each; for the criteria, see Supplementary material; criteria III 1–3 were combined for calculating the inter-observer reliability). This evaluation resulted in a Kendall’s coefficient of *W* = 0.86 and, accordingly, a good overall agreement. Subsequently, both observers evaluated all the ‘final’ video sequences using the assessment protocol. They were blinded to the educational programme of the respective participants. A video sequence was designated as being ‘final’ when a participant announced that the task was accomplished or, if this did not happen, the last training session conducted was used. From this final video we assessed the most successful attempt in the sequence of the last three repetitions of the goat showing the target behaviour (or what was closest to that).

We also assessed the inter-observer agreement for the final videos. Observers assessed 210 grades (seven elements of the assessment protocol for 30 goats each; elements I–VII in the Supplementary material). The observers agreed in 69% of the grades (with a difference of, at most, one grade) and deviated in their assessment by more than one, two, three and four grades in 21, 4.5, 5, and 0.5%, respectively. According to this agreement, the means of the two observers’ evaluation were used for further analysis.

### Statistical analysis

Data visualisation and analysis were carried out using R 4.2.2 (R Core Team 2022). The potential predictor variables were collected in the initial questionnaire. We further defined two groups of potential outcome variables. A first group included the data based on the video analysis (‘video analysis’), and a second included the data on self-assessment as reported in the second questionnaire that the participants filled out after the training period ended (‘self-assessment’; Table 2). For the statistical analysis, the grades of the two observers were averaged and then inversed such that a high value (6) reflected a good quality of the training and a low value (1) a failure of the training. To reduce the number of variables, and to avoid collinearity and multiple testing, we performed three Principle Component Analyses (PCA), one each for the group of potential predictors, for ‘video analysis’, and for ‘self-assessment’. The predictor ‘educational programme’ was not included in the PCA in order to be able to assess a potential difference between WORKSHOP GROUP versus BOOK GROUP more directly. Moreover, a first preliminary PCA of the predictor variables including the variable ‘educational programme’ had shown that this variable did co-vary only weakly with the other potential predictors and varied, in this sense, in an independent way (no collinearity). Most of the participants were female (n = 26). Therefore, the participants’ gender was not included in the further analyses. For every group of variables, the first two principal components (PCs) were used for further analyses (see *Results*).

The PCs were then used in linear models calculated using the function gls (R package ‘nlme’; Pinheiro *et al.* 2021). We used the first two PCs of the PCA for the ‘video analysis’ and the ‘self-assessment’ each as an outcome variable in four different models. ‘Educational programme’ (factor with two levels: WORKSHOP GROUP or BOOK GROUP; sum-contrasts) as well as the first two PCs of the PCA for the predictors (as continuous variables) and their interactions were used as the fixed effects.

Model assumptions were inspected by a visual analysis of residuals with a focus on normal distribution and homogeneity of variance. No major deviations from these assumptions were observed.

## Results

### Participants

**Figure 4.**
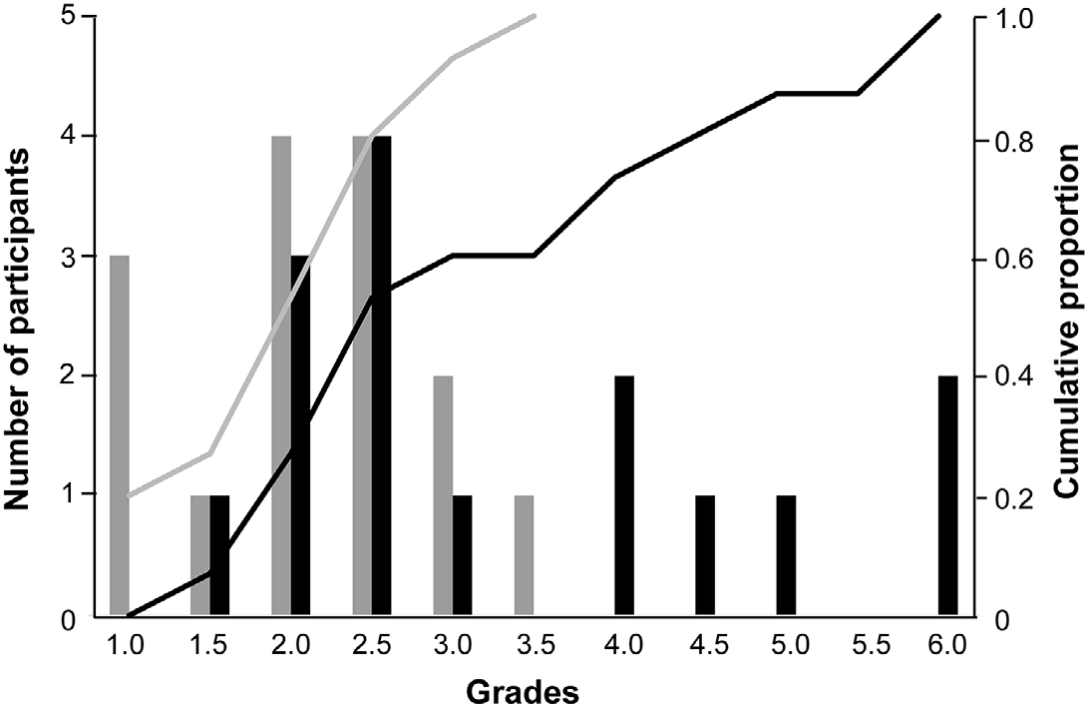
Distribution of grades 1–6 of ‘Goal achieved’ for the two educational programmes. 1 = equivalent to expert reference standard, 1.5 to 5.5 = deviations from expert reference standard and 6 = not showing the target behaviour. The grades were later inversed for the further statistical analysis such that high values reflected a high training quality. The bars represent the number of participants per grade (left Y-axis). The lines represent the cumulative proportion across grades (right Y-axis). WORKSHOP Group is represented in black, BOOK-GROUP in grey.

Participants of the WORKSHOP GROUP offered up a precise description of the target behaviour more often (n = 10/15) and created a training schedule more often (n = 10/15) than those in the BOOK GROUP (n = 3/15 and n = 4/15, respectively). The training task seemed both achievable and clear to n = 13/15 participants of the WORKSHOP GROUP, compared to n = 9 (clear) and n = 11/15 (achievable) in the BOOK GROUP. All participants used a primary reinforcer, and almost every participant used a secondary reinforcer (n = 27/30). The three participants training without a secondary reinforcer were all in the BOOK GROUP but one of them achieved the goal well. With one exception, all participants of the BOOK GROUP read the book completely (n = 8/15), or at least partly (n = 6/15). Approximately half of this group used additional resources to learn about animal training (n = 8/15) and thought that they could have trained more effectively with a workshop in advance (n = 7/15). All the WORKSHOP GROUP participants (n = 15) stated that the available time-slots were sufficient. In the BOOK GROUP n = 6/15 indicated that the available time-slots were not sufficient. Expert reference standard training took 34 min. In contrast, participants on the WORKSHOP GROUP spent 103 min on average to train their goats (range: 30–172 min), participants of the BOOK GROUP 148 min (range: 66–325 min). The target behaviour was completely achieved by only three of the participants, all of whom were in the WORKSHOP GROUP. Yet more participants came close to the expert reference standard (Figure 4).

### Assessment of training

To choose the number of principle components (PCs) from a PCA that will be considered in further analyses, a balance has to be reached between, on the one hand, the variance of the single PCs, their cumulative variance and, on the other, a small number of PCs and their ease of interpretation. Here, we reached a reasonable amount of cumulative proportion of variance explained with two PCs in each analysis and a solid interpretation when a threshold for the loadings was chosen at 0.3 (Table 3). In the PCA for the predictor variables, we found that the variables ‘age’, ‘prior education’, ‘experience’ (in years) and ‘experience with animals’ loaded strongly on the 1^st^ PC, which was considered to reflect overall ‘training experience’ correspondingly. On the 2^nd^ PC, observations reached high values with female goats (positive sign of loading) and participants that had no job with animals (negative sign). This PC is called ‘female goat’ here. For the ‘video analysis’, all original variables, except ‘jump’ and ‘respectfulness’, loaded strongly on the 1^st^ PC. The assessment of the seven elements did so with a positive, the time taken for training with a negative sign. This PC can be considered, therefore, as ‘objective success’. Observations with respectful goats that received weak grades for jumping reached high values on the 2^nd^ PC which can, accordingly, be interpreted as ‘self-control’. In the last PCA including the ‘self-assessment’ variables, ‘time-slots’, ‘implement content’, ‘content use helpful’, ‘goal self-assessment’, and ‘chin self-assessment’ loaded strongly on the 1^st^ PC. This PC accordingly reflects ‘subjective success’. On the 2^nd^ PC variables, ‘time-slots’, ‘former experience’ and ‘goal self-assessment’ predominantly loaded positively and ‘personal instruction’ negatively. This PC reflected ‘confidence’ of the participants.

**Table 3.** Results of the Principle Component Analysis conducted on the potential predictor variables, variables from the video analysis, and variables from the self-assessment. The first two components for each analysis are shown including the proportion of explained variance, cumulative proportion of explained variance and the loadings. Loadings > 0.3 in bold. For the description of the variables, see Table 2

Predictors (initial questionnaire)			Video analysis			Self-assessment		
	1^st^ PC	2^nd^ PC		1^st^ PC	2^nd^ PC		1^st^ PC	2^nd^ PC
	Training experience	Female Goat		Objective success	Self-Control		Subjective success	Confidence
Proportion of variance	0.450	0.205		0.505	0.158		0.408	0.192
Cumulative proportion	0.450	0.655		0.505	0.663		0.408	0.600
Sex of goat	–0.006	**0.719**	Session	**–0.323**	–0.103	Time slots	**0.359**	**0.457**
Age	**0.410**	–0.251	Duration	**–0.320**	–0.053	Former experience	–0.220	**0.586**
Job with animal	0.004	**–0.622**	Jump	0.244	**–0.492**	Pers. instruction	0.130	**–0.464**
Prior education	**0.493**	0.180	Chin	**0.395**	0.117	Implement content	**0.500**	–0.006
Experience years	**0.538**	0.027	Touch	**0.365**	–0.247	Content use helpful	**0.451**	–0.154
Experience with animals	**0.547**	0.011	Standing still	**0.349**	0.172	Goal self-Assessment	**0.365**	**0.437**
			Trait trainer	**0.359**	0.302	Chin self-assessment	**0.468**	–0.132
			Respectfulness	–0.023	**0.736**			
			Goal achieved	**0.439**	–0.087			

**Figure 5.**
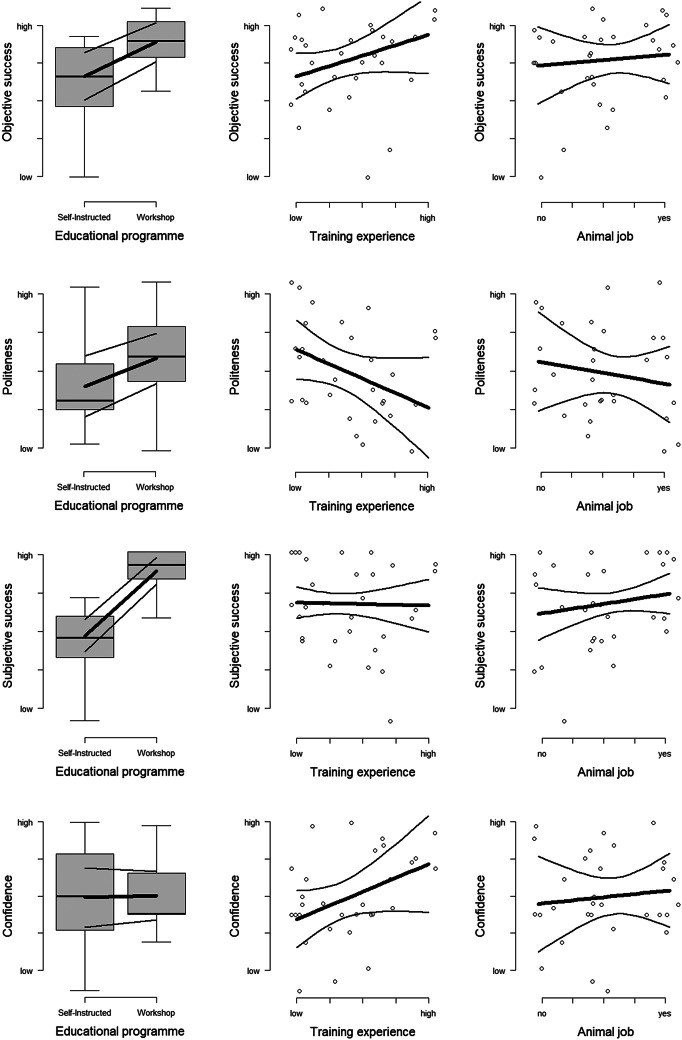
Outcome variables ‘objective success’, ‘self-control’, ‘subjective success’ and ‘confidence’ plotted against the predictors ‘educational programme’, ‘training experience’, and ‘female goat’, reflecting the results of the Principal Component Analysis (PCA) presented in Table 3. All figures including model estimates (thick lines) and 95% confidence intervals (thin lines).

Participants of the WORKSHOP GROUP reached higher values of ‘objective success’ than those of the BOOK GROUP (*F*_1,26_ = 5.37; *P* = 0.030; Figure 5). On average, ‘objective success’ was higher for participants with higher ‘training experience’ although this effect could not be supported statistically (*F*_1,26_ = 2.60; *P* = 0.12; Figure 5). Statistical support for all remaining effects was lower (all *P* > 0.29).

‘Self-control’ was somewhat higher in goats trained by the WORKSHOP GROUP than in goats trained by the BOOK GROUP, although this effect could not be supported statistically (*F*_1,26_ = 2.17; *P* = 0.15; Figure 5). Goats were less respectful on average with participants who had higher training experience (*F*_1,26_ = 3.04; *P* = 0.095). All other effects had weaker statistical support (all *P* > 0.55).

Participants of the WORKSHOP GROUP reached higher values of ‘subjective success’ (*F*_1,26_ = 41.63; *P* < 0.0001; Figure 5). All other effects had no statistical support (all *P* > 0.30).

There was no average effect on ‘confidence’ by ‘educational programme’ (*F*_1,26_ = 0.01; *P* = 0.91; Figure 5). Yet, higher ‘training experience’ was related to higher ‘confidence’ (*F*_1,26_ = 2.98; *P* = 0.098). All other effects had low statistical support (all *P* > 0.45).

‘Objective success’ and ‘subjective success’ correlated positively (Spearman’s rank correlation: rho= 0.573; *P* < 0.0001).

## Discussion

The aim of this study was to evaluate the influence of trainer education on success in animal training. The assessment of training success confirmed our hypothesis that participants educated in a two-day workshop (WORKSHOP GROUP) had a greater ‘objective success’ than participants of the BOOK GROUP. Accordingly, goats trained by this group achieved the target behaviour more closely. This is in agreement with LaFollette *et al.* (2020) who evaluated different educational programmes to implement rat tickling in an animal facility as refinement technique. Their study showed that participants with hands-on training supplementing online education learned more effectively than participants trained purely online. Workshops, as used in the current study, are an active learning method, which is known to lead to good learning outcomes (Lim *et al.* 2019). Furthermore, participants of the WORKSHOP GROUP could define the target behaviour more accurately at the end of the training than participants of the BOOK GROUP. Such an accurate definition is needed to develop a structure for training. Accordingly, the specific instructions participants received during the workshop could have led to more structured and, therefore, successful training of the goats. This is also seen in the higher number of participants creating a training schedule before training their goats in the WORKSHOP GROUP compared with the BOOK GROUP.

The participants’ prior training experiences had only a minor yet positive influence on the ‘objective success’. This indicates that specific instruction, or further education for specific target behaviours are still helpful, even when knowledge and experience in animal training is present. Previous studies only speculated on the influence of trainers’ experience on training success, as mentioned in scent detection training for dogs (Johnen *et al.* 2013), or in positive reinforcement training on monkeys (Fischer & Wegener 2018). To compare the efficiency of different training methods, the effect of animal trainers has to be evaluated. However, it is difficult to assess the quality of previous training experience as part of training skills. In our study, only the quantity of training experience was self-reported by the participants. Subjective biases can, therefore, occur and it is possible that participants overestimated their own experience. Such an assessment would be eased if there were consistent educational programmes or certificates for animal training.

‘Subjective success’ was also higher in the WORKSHOP GROUP than in the BOOK GROUP. Furthermore, there was a positive correlation between ‘objective’ and ‘subjective success’. This indicates that participants in both groups tended to self-assess their training success in accordance with their ‘objective success’. Self-assessment may help to facilitate skills, as seen in a study about veterinary students learning basic surgical skills (Tobias & Bailey 2020).

As there are no evidence-based protocols in the literature to assess the training success of animal trainers objectively, we developed a non-dichotomous assessment to analyse video sequences of training sessions. With this, we could also reflect partial achievement of the target behaviour. Using this protocol, the inter-observer agreement with the assessment protocol had a strong agreement (0.86; Landis & Koch 1977) indicating that the preceding observer training was sufficient and that the assessment protocol described the behaviour adequately. The protocol did not include the frequency nor the timing of the rewards, given the technical limitations of the videos and the necessary training experience of the observers. However, we hope to address this aspect in future research.

Only three WORKSHOP GROUP participants achieved the training goal completely. It is apparent that a two-day workshop did not adequately equip participants with the necessary skills to accomplish the training goal. This is in agreement with Lukas *et al.* (1998) where students educated through a book and practical sessions, had to train different species of zoo animals for specific target behaviours. Some students successfully trained the target behaviors in full, while others achieved partial success. Participants of the BOOK GROUP invested more time in training their goats, and were less successful. Furthermore, ‘duration’ and ‘time’ loaded with negative signs on ‘objective success’. This indicates that less training time is needed when the trainer is more skilled. Accordingly, VT trained the expert reference standard goat much faster compared to the participants (in only 34 min). Hence, further research is necessary to understand the optimal intensity of animal training education required for trainers to effectively implement training methods. Johnen *et al.* (2013) reviewed studies about scent detection with dogs and described a wide variation in the duration of dog training between seven days and 16 months. Concerning animal research, this wide range has to be considered when planning the duration of a training period prior to the onset of an experiment.

According to LaFollete *et al.* (2020), rat tickling can reduce stress in both rats and humans. Husbandry training could have the same effect. One outstanding feature of this study was that in their oral feedback all the participants emphasised how much they enjoyed training the goats, regardless of their training success. Furthermore, our animal caretakers reported that handling of the goats was easier and less stressful for them and the animals after the two-week training period. Their statements and our own impressions suggest that the training furthered a positive human-animal relationship. According to Rault *et al.* (2020), a positive human-animal-relationship is a key for animal welfare and can be achieved, amongst other things, through positive reinforcement training. Therefore, the results of this study suggest that positive reinforcement training, independent of the target behaviour, has a high potential to improve animal welfare. However, the more precisely a voluntary behaviour is trained, the less restraint is needed. Therefore, more successful husbandry training may have a greater contribution to animal welfare regarding stress reduction. In our study, we were able to show successful husbandry training to be based on intensive education in positive reinforcement training and this training requires time.

### Study limitations

In video analyses, there is always a risk of bias due to the observer’s conscious or unconscious predispositions. Disguising participants was not possible due to the importance of gestures and body positions for evaluation. Therefore, body type (Puhl & Brownell 2001), voice (Mileva *et al.* 2018) or age (Voss *et al.* 2018) could have had an influence on the observer’s assessment. However, the observers were blinded for the educational programme.

All participants trained the goats in their free time. Personal resources, travel time to BfR, or other factors may have influenced the number of training sessions each participant undertook. However, only participants of the BOOK GROUP stated that the allotted time-slots were insufficient.

### Animal welfare implications

Husbandry training as a refinement measure for animals has been shown to have a high potential to improve animal welfare. Specifically trained farm animals, which participate voluntarily in a procedure, require less restraining. Our study focused on the education of animal trainers as one potential key for successful husbandry training. The results show that with intensive education, implementation of animal training can serve as a refinement measure. Furthermore, we want to highlight the potential of animal training as enrichment as well as a tool to improve a positive human-animal relationship. We see a high potential of further education in husbandry training and its implementation as a refinement measure to increase the welfare of the animals concerned.

## Conclusion

This study shows benefits of hands-on training in husbandry training compared with self-instruction. To implement husbandry training effectively as refinement in farm animals in biomedical research, we highly suggest specific education on animal training. Hands-on and case-based training with experienced trainers as with our workshop is recommended. A protocol to assess training success can be useful to guide the animal trainer in identifying potential for improvement in their training skills.

## Supporting information

Meier et al. supplementary material Meier et al. supplementary material

## Supplementary material

The supplementary material for this article can be found at http://doi.org/10.1017/awf.2023.94.

## References

[r1] Bailey J 2018 Does the stress of laboratory life and experimentation on animals adversely affect research data? A critical review. Alternatives to Laboratory Animals 46: 291–305. 30488713 10.1177/026119291804600501

[r2] Balcombe JP, Barnard ND and Sandusky C 2004 Laboratory routines cause animal stress. Journal of the American Association for Laboratory Animal Science 43(6): 42–51. https://www.ingentaconnect.com/content/aalas/jaalas/2004/00000043/00000006/art00009 15669134

[r3] Bansal A and Aggarwal S 2014 Impact of ’Workshop’ on the performances of first professional medical students in Physiology. Biomedical Research (India) 25: 127–131.

[r4] Bloomsmith M, Neu K, Franklin A, Griffis C and McMillan J 2015 Positive reinforcement methods to train chimpanzees to cooperate with urine collection. Journal of the American Association of Laboratory Animal Science 54(1): 66–69. PMC4311744 25651093

[r5] Colahan H and Breder C 2003 Primate training at Disney’s Animal Kingdom. Journal of Applied Animal Welfare Science 6(3): 235–246. 10.1207/s15327604jaws0603_08 14612271

[r6] Do Vale GT, Leoni D, Sousa AH, Gonzaga NA, Uliana DL, La Gata DC, Resstel LB, Padovan CM and Tirapelli CR 2020 Acute restraint stress increases blood pressure and oxidative stress in the cardiorenal system of rats: a role for AT(1) receptors. Stress 23(3): 328–337. 10.1080/10253890.2019.1675627 31583926

[r7] European Commission 2023 Summary report on the statistics on the use of animals for scientific purposes in the member states of the European Union and Norway in 2020. EC: Brussels, Belgium. https://circabc.europa.eu/ui/group/8ee3c69a-bccb-4f22-89ca-478277e35de7c63/library/10ad28d6-e17e-4367-b459-20883402cfcc/details

[r8] Ferguson DL and Rosales-Ruiz J 2001 Loading the problem loader: the effects of target training and shaping on trailer-loading behavior of horses. Journal of Applied Behavioural Analysis 34(4): 409–423. 10.1901/jaba.2001.34-409 PMC1284337 11800182

[r9] Fernandez EJ 2020 Training petting zoo sheep to act like petting zoo sheep: An empirical evaluation of response-independent schedules and shaping with negative reinforcement. Animals 10(7): 1122. 10.3390/ani10071122 32630257 PMC7401582

[r10] Fernandez EJ 2022 Training as enrichment: A critical review. Animal Welfare 31 (1): 1–12. 10.7120/09627286.31.1.001

[r11] Fernandez EJ and Martin AL 2021 Animal training, environmental enrichment, and animal welfare: A history of behavior analysis in zoos. Journal of Zoological and Botanical Gardens 2(4): 531–543. https://www.mdpi.com/2673-5636/2/4/38

[r12] Fischer B and Wegener D 2018 Emphasizing the ’positive’ in positive reinforcement: using nonbinary rewarding for training monkeys on cognitive tasks. Journal of Neurophysiology 120(1): 115–128. 10.1152/jn.00572.2017 29617217

[r13] Gamer M, Lemon J, Fellows I and Singh P 2019 *irr: Various Coefficients of Interrater Reliability and Agreement. R package version 0.84.1.* https://www.r-project.org

[r14] Hamernik DL 2019 Farm animals are important biomedical models. Animal Frontiers 9(3): 3–5. 10.1093/af/vfz026 PMC6951888 32002256

[r15] Hawthorne WJ 2022 World first pig-to-human cardiac xenotransplantation. Xenotransplantation 29(1): e12733. 10.1111/xen.12733 35172036 PMC9286822

[r16] Johnen D, Heuwieser W and Fischer-Tenhagen C 2013 Canine scent detection—Fact or fiction? Applied Animal Behaviour Science 148(3): 201–208. 10.1016/j.applanim.2013.09.002

[r17] Jonholt L, Bundgaard CJ, Carlsen M and Sorensen DB 2021 A case study on the behavioural effect of positive reinforcement training in a novel task participation test in Gottingen mini pigs. Animals (Basel) 11(6). 10.3390/ani11061610 PMC8229723 34072458

[r18] Lafollette MR, Cloutier S, Brady CM, O’Haire ME and Gaskill BN 2020 Changing human behavior to improve animal welfare: A longitudinal investigation of training laboratory animal personnel about heterospecific play or ’rat tickling’. Animals (Basel) 10(8). 10.3390/ani10081435 PMC7459457 32824457

[r19] Landis JR and Koch GG 1977 The measurement of observer agreement for categorical data. Biometrics 33(1): 159–174. 10.2307/2529310 843571

[r20] Laule G, Bloomsmith M and Schapiro S 2003 The use of positive reinforcement training techniques to enhance the care, management, and welfare of primates in the laboratory. Journal of Applied Animal Welfare Science 6: 163–173. 10.1207/S15327604JAWS0603_02 14612265

[r21] Leidinger C, Herrmann F, Thöne-Reineke C, Baumgart N and Baumgart J 2017 Introducing clicker training as a cognitive enrichment for laboratory mice. Journal of Visualised Experiments 121. 10.3791/55415 PMC5408971 28287586

[r22] Lim J, Ko H, Yang Ji W, Kim S, Lee S, Chun M-S, Ihm J and Park J 2019 Active learning through discussion: ICAP framework for education in health professions. BMC Medical Education 19(1): 477. 10.1186/s12909-019-1901-7 31888595 PMC6937678

[r23] Lomb J, Mauger A, Von Keyserlingk MAG and Weary DM 2021 Effects of positive reinforcement training for heifers on responses to a subcutaneous injection. Journal of Dairy Science 104(5): 6146–6158. 10.3168/jds.2020-19463 33685711

[r24] Lukas K, Marr M and Maple T 1998 Teaching operant conditioning at the zoo. Teaching of Psychology 25: 112–116. 10.1207/s15328023top2502_7

[r25] Melfi VA, Dorey NR and Ward SJ 2020 Zoo Animal Learning and Training, First Edition. John Wiley & Sons Ltd: London, UK. 10.1002/9781118968543

[r26] Mileva M, Tompkinson J, Watt D and Burton AM 2018 Audiovisual integration in social evaluation. Journal of Experimental Psychology: Human Perception and Performance 44(1): 128–138. 10.1037/xhp0000439 28481565

[r27] Miotto HC, Camargos FR, Ribeiro CV, Goulart EM and Moreira Mda C 2010 Effects of the use of theoretical versus theoretical-practical training on CPR. Arquivos Brasileiros de Cardiologia 95(3): 328–331. 10.1590/s0066-782x2010005000104 20721520

[r28] Paredes-Ramos P, Diaz-Morales JV, Espinosa-Palencia M, Coria-Avila GA and Carrasco-Garcia AA 2020 Clicker training accelerates learning of complex behaviors but reduces discriminative abilities of Yucatan miniature pigs. Animals: an open access journal from MDPI 10(6): 959. 10.3390/ani10060959 32486472 PMC7341331

[r29] Pinheiro J, Bates D, Debroy S and Sarkar D 2021 *nlme: Linear and Nonlinear Mixed Effects Models.* R Core Team. https://svn.r-project.org/R-packages/trunk/nlme/

[r30] Poole T 1997 Happy animals make good science. Laboratory Animals 31(2): 116–124. 10.1258/002367797780600198 9175008

[r31] Puhl R and Brownell KD 2001 Bias, discrimination, and obesity. Obesity Research 9(12): 788–805. 10.1038/oby.2001.108 11743063

[r32] R Core Team 2022 *R: A language and environment for statistical computing.* R Foundation for Statistical Computing. https://www.R-project.org/

[r33] Rault JL, Waiblinger S, Boivin X and Hemsworth P 2020 The power of a positive human-animal relationship for animal welfare. Frontiers in Veterinary Science 7: 590867. 10.3389/fvets.2020.590867 33240961 PMC7680732

[r34] Rosenberger G, Dirksen G, Gründer H and Stöber M 1977 Die Klinische Untersuchung des Rindes, Volume Three. Verlag Paul Parey: Germany. [Title translation: The clinical examination of cattle]

[r35] Russell WMS and Burch RL 1959 The Principles of Humane Experimental Technique. Methuen: London, UK.

[r36] Savastano G, Hanson A and McCann C 2003 The development of an operant conditioning training program for New World primates at the Bronx Zoo. Journal of Applied Animal Welfare Science 6(3): 247–261. 10.1207/S15327604JAWS0603_09 14612272

[r37] Schapiro S, Bloomsmith M and Laule G 2003 Positive reinforcement training as a technique to alter non-human primate behavior: Quantitative assessments of effectiveness. Journal of Applied Animal Welfare Science 6: 175–187. 10.1207/S15327604JAWS0603_03 14612266

[r38] Schapiro SJ, Perlman JE and Boudreau BA 2001 Manipulating the affiliative interactions of group-housed rhesus macaques using positive reinforcement training techniques. American Journal of Primatology 55(3): 137–149. 10.1002/ajp.1047 11746277

[r39] Sevenich‐Macphee M 2019 Integrating training into animal husbandry. *Zoo Animal Learning and Training.* 10.1002/9781118968543.ch8

[r40] Skinner BF 1951 How to teach animals. Scientific American 185(6): 26–29.

[r41] Slater C and Dymond S 2011 Using differential reinforcement to improve equine welfare: Shaping appropriate truck loading and feet handling. Behavioural Processes 86(3): 329–339. 10.1016/j.beproc.2011.02.001 21310219

[r42] Theby V 2020 Verstärker verstehen:Über den Einsatz von Belohnung im Hundetraining, Fifth Edition. Kynos: Sheffield, UK. [Title translation: Understanding reinforcement]

[r43] Tobias KM and Bailey MR 2020 Veterinary student self-assessment of basic surgical skills as an experiential learning tool. Journal of Veterinary Medicine Education 47(6): 661–667. 10.3138/jvme.2018-0004 32053054

[r44] Voss P, Bodner E and Rothermund K 2018 Ageism: The relationship between age stereotypes and age discrimination. In: Ayalon L and Tesch-Römer C (eds) Contemporary Perspectives on Ageism pp 11–31. Springer International Publishing: London, UK. 10.1007/978-3-319-73820-8_2

[r45] Yang HY, Galang KG, Gallegos A, Ma BW and Isseroff RR 2021 Sling training with positive reinforcement to facilitate porcine wound studies. JID Innovations 1(2): 100016. 10.1016/j.xjidi.2021.100016 35024682 PMC8669512

[r46] Yardimci M, Sahin EH, Cetingul IS, Bayram I, Aslan R and Sengor E 2013 Stress responses to comparative handling procedures in sheep. Animal 7(1): 143–150. 10.1017/S1751731112001449 23031646

