# Supplementary material for: Why workshops work: Examining the efficacy of training trainers to train goats
Source: Anim Welf. 2023 Nov 21;32:e76. doi: 10.1017/awf.2023.94 (PMC10955182; doi:10.1017/awf.2023.94)
Supplement: Meier et al. supplementary material [file S0962728623000945sup001.pdf]

Assessment Protocol for Video Analysis

Here, the assessment protocol for analysing the videos including all definitions is reported. Seven elements (I – VII) were assessed based on a total of 14 criteria. The observers had a scoring sheet for each video on which they marked behaviour that was shown by the goats and the participants with a cross and noted additionally durations if mentioned in the definitions. Scores and grades were not shared with the observers. The grading was done afterwards by JM based on the crosses (and durations) noted by the observers.

I. Goat on platform

		Grade
a	**Goat jumps immediately (≤ 2 sec) after request/ command on platform**	**1**
b	**Goat jumps immediately (≤ 2 sec) without request/ command on platform**	**2**
c	**Goat jumps after delay / luring (> 2 sec.) on platform**	**3**
d	**Goat climbs platform / rung with foreleg before jumping on platform**	**4**
e	**Goat climbs platform / rung with foreleg without jumping on platform**	**5**
f	**Goat does not interact with nor jumps on platform**	**6**

II: Chin on Hand

The overall grade of element II was calculated as the arithmetic mean of the three criteria (II. 1. - 3.).

1. Chin target 1: How fast does the goat lay its chin on the participant's hand?

			Grade
a	**The goat lays its chin immediately on the presented hand** (seems to perceive hand as a signal)		**1**
b	**Goat shows other behaviour** (sniffling, looking at trainer…) **before laying its chin on the hand**	1 – <3 sec	**2**
		≥ 3 sec	**3**
c	**Goat lays its chin on the hand only if lured with treats**		**4**
d	**Trainer lays actively their hand under the goat's chin**		**2**
e	**Goat does not show the behaviour "chin on hand"**		**6**

2. Chin target 2: position of feeding

			Grade
a	**Goat lays its chin on the hand without being fed**	≥ 3 sec	**1**
		1 – < 3 sec	**2**
b	**Goat lays its chin on the hand while being constantly fed / feeding hand stays at target hand and goat**		**4**

3. Chin target 3: How still does the goat keep its chin on the hand?

			Grade
a	**Goat stays still, hand does not move**	≥ 2 sec	**1**
		1 – < 2 sec	**2**
b	**Goat moves head, but stays in contact with hand, hand does not move**		**3**
c	**Goat moves head, but stays in contact with hand, hand follows the goat's movement**		**4**
d	**Goat does not have constant but repeated contact to the hand**		**5**
f	**Goat has contact with hand only when being fed/ moves head as soon as there is no treat**		**6**

III. Touch of the neck

The overall grade of element III was calculated as the arithmetic mean of the three criteria (III. 1. – 3).

1. Who touches the goat's neck?

			Grade
a	**Second person**	≥ 5 sec	**1**
		< 5 sec	**2**
b	**Trainer**	≥ 5 sec	**3**
		< 5 sec	**4**

2. When does the goat react to the touch of the neck?

a	**Goat reacts on touch when it is not fed**		3
b	**Goat reacts on touch even when it is fed**		5

3. How does the goat react to the touch of the neck?

a	**Goat does not react on touch and stays still**		1
b	**Goat moves not away while being touched, but before/after touch**		3
c	**Goat looks at the hand, but does not move its body away from the hand**		4
d	**Goat moves body away / makes defensive movement** (e.g moves forehead to hand)		6
f	**Touch of the neck is not performed**		6

IV. Standing still

The overall grade of element IV was calculated as the arithmetic mean of the two criteria (IV. 1. & 2).

1. How still does the goat stand during task?

			Grade
a	**Goat stands still with all four limbs on the platform (without shifting of weight)**	≥ 5 sec	**1**
		< 5 sec	**2**
b	**Goats shifts its weight between limbs**		**3**
c	**Goats "bobs" / makes steps / lifts a limb during target behaviour**		**5**

2. Time: How long does the goat keep its chin on the hand (without feed)

			Points
a	**For how long does the goat stand still in goal position including touch of the neck?**	≥ 2 sec	**+2**
		< 2 sec	**+1**
b	**For how long does the goat keep its chin on the hand (without feed)**	≥ 3 sec	**+2**
		< 3 sec	**+1**
c	**How long is the goat standing on all four limbs** (without shifting of weight) **on platform**	≥ 5 sec	**+2**
		< 5 sec	**+1**

Grading scale table: Points for element IV, (criteria 1 and 2) were added and then assigned a grade as follows:

Points (IV)	Grade
6	1
5	2
4 / 3	3
2	4
1	5
0	6

V. Behaviour of participant (Trainer Skills)

1. How is the target behaviour stopped?

		Points
a	**Trainer ends behaviour with removing the target hand**	+1
b	**Goat ends behaviour with lifting the head from the hand/ looking away/ turning body/ walking away from trainer**	0
c	**Goat ends behaviour with jumping from the platform**	-1

2. Trainer

		Points
a	**Person uses primary reinforcer** (= feed)	+1
b	**Person uses secondary reinforcer** (Clicker / Marker word)	+1
c	**Primary reinforcer (feed) is used selectively**	+1
d	**Secondary reinforcer is used before primary reinforcer**	+1
e	**Reinforcer is constantly used** (while the chin lays on the hand)	- 1
f	**Stands still in one position**	+1
g	**Has a neutral position (in particular position of hands), to which they return after feeding**	+1
h	**Changes their position (in contrast to "f")**	- 1
i	**Feeding point is at the hand where the goat should lay its chin on** (i.e. feed goes to the goat and not vice versa)	+1
j	**Feeding hand moved away from target hand and goat** (and comes back)	+1

3. Criterion for rewarding: rewarding/ feed shows specific aspects listed

		Points
a	**A specific criterion for rewarding is observable**<br>Please note the criterion: 1 (Goat touches hand with chin), 2 (Chin on hand, standing still) 3 (other -> please note)	+1
b	**The criterion remains constant**	+1
c	**No rewarding criterion is observable**	- 1
d	**Trainer rewards/ „clicks" for unwanted behaviour** (e.g. lifting head from the hand, moving head, not standing still…)	- 1

Grading scale table: Points for element V, (criteria 1 – 3) were added and then assigned a grade as follows:

Points (V)	Grade
11 / 10	1
9 / 8	2
7 / 6	3
5 / 4	4
3 / 2	5
1 / ≤ 0	6

VI. Respectfulness

		Grade
a	**Goat is actively searching for feed**	5
b	**Goat stands with its forelimbs on the edge of the table**	4
	**None of the above**	1

VII. Overall grade (Training goal was completely accomplished)

		Points
a	**Jumping on platform (immediately)**	+1
b	**Chin on hand (without feed) (> 3 sec.)**	+1
c	**Tolerating the touch of the neck (> 3 sec)**	+1
d	**Goat stays still, hand does not move (≥ 2 sec)**	+1

Grading scale table: Points for element VII were added and then assigned a grade as follows:

Points (VII)	Grade
4	1
3	2
2	3
1	4
-	5
0	6
